# Treatment with Zinc is Associated with Reduced In-Hospital Mortality Among COVID-19 Patients: A Multi-Center Cohort Study

**DOI:** 10.21203/rs.3.rs-94509/v1

**Published:** 2020-10-26

**Authors:** Jennifer A. frontera, Joseph O. Rahimian, Shadi Yaghi, Mengling Liu, Ariane Lewis, Adam de Havenon, Shraddha Mainali, Joshua Huang, Erica Scher, Thomas Wisniewski, Andrea B. Troxel, Sharon Meropol, Laura J. Balcer, Steven L. Galetta

**Affiliations:** NYU Langone Health; University of Utah Health; Ohio State University Foundation: The Ohio State University; NYU Langone Health

**Keywords:** Zinc, ionophore, treatment, mortality, COVID-19, SARS-CoV-2

## Abstract

**Background:**

Zinc impairs replication of RNA viruses such as SARS-CoV-1, and may be effective against SARS-CoV-2. However, to achieve adequate intracellular zinc levels, administration with an ionophore, which increases intracellular zinc levels, may be necessary. We evaluated the impact of zinc with an ionophore (Zn+ionophore) on COVID-19 in-hospital mortality rates.

**Methods:**

A multicenter cohort study was conducted of 3,473 adult hospitalized patients with reverse-transcriptase-polymerase-chain-reaction (RT-PCR) positive SARS-CoV-2 infection admitted to four New York City hospitals between March 10 through May 20, 2020. Exclusion criteria were: death or discharge within 24h, comfort-care status, clinical trial enrollment, treatment with an IL-6 inhibitor or remdesivir. Patients who received Zn+ionophore were compared to patients who did not using multivariable time-dependent cox proportional hazards models for time to in-hospital death adjusting for confounders including age, sex, race, BMI, diabetes, week of admission, hospital location, sequential organ failure assessment (SOFA) score, intubation, acute renal failure, neurological events, treatment with corticosteroids, azithromycin or lopinavir/ritonavir and the propensity score of receiving Zn+ionophore. A sensitivity analysis was performed using a propensity score-matched cohort of patients who did or did not receive Zn+ionophore matched by age, sex and ventilator status.

**Results:**

Among 3,473 patients (median age 64, 1947 [56%] male, 522 [15%] ventilated, 545[16%] died), 1,006 (29%) received Zn+ionophore. Zn+ionophore was associated with a 24% reduced risk of in-hospital mortality (12% of those who received Zn+ionophore died versus 17% who did not; adjusted Hazard Ratio [aHR] 0.76, 95% CI 0.60–0.96, P=0.023). More patients who received Zn+ionophore were discharged home (72% Zn+ionophore vs 67% no Zn+ionophore, P=0.003) Neither Zn nor the ionophore alone were associated with decreased mortality rates. Propensity score-matched sensitivity analysis (N=1356) validated these results (Zn+ionophore aHR for mortality 0.63, 95%CI 0.44–0.91, P=0.015). There were no significant interactions for Zn+ionophore with other COVID-19 specific medications.

**Conclusions:**

Zinc with an ionophore was associated with increased rates of discharge home and a 24% reduced risk of in-hospital mortality among COVID-19 patients, while neither zinc alone nor the ionophore alone reduced mortality. Further randomized trials are warranted.

## Summery

In this study of 3,473 hospitalized COVID-19 patients, treatment with zinc and an ionophore was associated with a 24% reduced risk of in-hospital mortality in multivariable Cox regression analysis. A sensitivity analysis in a propensity score-matched cohort supported this finding.

## Background

Efforts to identify therapeutic interventions to ameliorate the impact of COVID-19 have resulted in over 200 ongoing clinical trials, evaluating a wide spectrum of medications including antivirals, antimalarials, immunomodulators, plasma-based therapies, stem cell therapies, anticoagulants and vitamin/mineral supplementation(1). One potential therapy of interest is zinc, which has a range of immune-modulating effects including regulation of leukocyte function and tempering of inflammatory responses(2, 3). Pre-clinical studies have shown that zinc acts to impair replication of RNA viruses, including SARS-CoV-1, through direct inhibition of RNA-dependent RNA polymerase(4). It is postulated that zinc may similarly inhibit SARS-CoV-2 replication, though little data currently exists to support this hypothesis(5, 6).

Under normal physiological conditions, intracellular levels of zinc are maintained at low and possibly subtherapeutic levels by metallothioneins(7). In order to effectively increase intracellular zinc levels, co-administration with an ionophore is necessary. Ionophores are lipid soluble compounds that act by promoting intracellular transport of non-lipid soluble zinc, independent of zinc binding proteins present in the plasma membrane. Several compounds have been identified as zinc ionophores including pyrithione(4), pyrrolidine dithiocarbamate(4), hinokitiol(8), resveratrol(9), plant polyphenols such as quercetin and epigallocatechin-gallate(10), chloroquine and hydroxychloroquine(11). One randomized trial of hydroxychloroquine in non-hospitalized adults with early COVID-19 examined a subgroup of 63 patients (15% of the analyzed cohort of 423) who received zinc(12). Though there was a 3% reduction in disease severity among those who received both zinc and hydroxychloroquine, this difference did not meet statistical significance in this small subgroup.

Based on the putative antiviral effect of zinc in preclinical studies of SARS-CoV-1 (4), we hypothesized *a priori* that administration of zinc along with an ionophore would be efficacious in the treatment of patients with SARS-CoV-2 infection. Therefore, zinc administration was suggested by hospital pharmacists among patients who were already receiving the ionophore hydroxychloroquine as part of a hospital-wide guideline for the management of patients with COVID-19.

The primary aim of this study was to compare rates of in-hospital mortality among adult hospitalized COVID-19 patients who received zinc plus an ionophore (Zn + ionophore) to those who did not.

## Methods

### Study Design and Participants

A multi-center cohort study of consecutive hospitalized patients admitted between March 10, 2020 and May 20, 2020 was conducted. Inclusion criteria were: aged ≥ 18 years, hospital admission, and reverse-transcriptase-polymerase-chain-reaction (RT-PCR) confirmed SARS-CoV-2 infection. Exclusion criteria were: death or discharge < 24 hours from admission, comfort care status, participation in a clinical trial (drug or convalescent plasma), treatment with an interleukin-6 inhibitor/modulator (including clazakizumab, tocilizumab, or sarilumab), treatment with Remdesivir, treatment with zinc or hydroxychloroquine for < 24 hours, or treatment in an emergency department or outpatient setting only.

A sensitivity analysis was performed in a propensity score-matched cohort of patients who did or did not receive Zn + ionophore, matched by age, sex, and invasive mechanical ventilation status.

### Setting

This study included patients admitted to four NYU Langone Hospitals located in Manhattan, Brooklyn and Mineola, New York. All four hospitals utilize the same electronic medical record ([EMR], Epic) and information technology center, and have integrated clinical protocols for patient management. This study was approved with a waiver of authorization and informed consent by the NYU Grossman School of Medicine Institutional Review Board based on the low risk to patients.

### Data Collection

Demographics, past medical history, medication utilization and in-hospital outcomes (in-hospital mortality, discharge disposition, ventilator days and hospital length of stay) were extracted from the EMR. The maximum recorded Sequential Organ Failure Assessment (SOFA) Score was used to assess severity of illness and has been shown to be predictive of organ failure and in-hospital mortality(13–15). Specific in-hospital complications including neurological events (e.g. stroke, toxic/metabolic encephalopathy, hypoxic/ischemic brain injury, seizures), and acute respiratory failure requiring invasive mechanical ventilation were ascertained via manual chart review following the Global Consortium Study-NeuroCOVID study protocol and data dictionary(16). The in-hospital complication of acute renal failure was assessed by EMR data query based on ICD-10 diagnoses.

### Exposure

Patients were coded as having received Zn + ionophore if the combination of zinc and hydroxychloroquine were administered together at any time during hospitalization for a minimum duration of one day. Data on the dose, route, duration of administration and time from admission to first dose were extracted. A time-dependent Zn + ionophore covariate was used to account for “immortal time bias”, which can occur when an event is observed more frequently in patients who survive long enough to be diagnosed or treated with a particular medication(17). This methodology also allowed us to account for the time from admission to exposure to medication when predicting the hazard of in-hospital death.

### Medication Utilization

A health system-wide inpatient COVID-19 treatment algorithm was developed by the hospital infection control and infectious diseases departments and was iteratively revised over the study time frame (see Supplemental Methods). This guideline broadly followed Infectious Diseases Society of America Guidelines for the treatment and management of COVID-19(18). Hospital guidelines for the treatment for COVID-19 patients at the time (instituted in March, 2020) recommended administration of hydroxychloroquine (400 mg BID for one day then 200 mg BID for four days) in patients whose oxygen saturation was < 94% on room air and whose QTc interval was < 500 ms. Zinc sulfate (220 mg [50 mg Zinc] PO once or twice daily) was suggested in combination with the ionophore hydroxychloroquine(11) beginning March 26, 2020, because achieving elevated intracellular levels with zinc alone is difficult(19). Zinc was therefore administered at the same time as hydroxychloroquine, typically for four days or until discharge. Zinc could be prescribed for longer courses at the discretion of the treating physician. During the timeframe of this study (prior to publication of the RECOVERY trial(20)), corticosteroids could be considered in patients with bilateral opacities on chest imaging and a PaO2/FiO2 < 250 mmHg, as long as the patient did not have an active bacterial infection and was not immunosuppressed. Corticosteroids could also be used for other indications, such as COPD exacerbation, refractory septic shock or suspected adrenal insufficiency. A protocol of early proning was promoted for patients prior to intubation.

### Study Outcomes

The primary outcome was in-hospital death. To avoid time to event bias among patients who were discharged, a dummy variable of 75 days was used as the event time for right censored patients who were not dead or discharged to hospice. Seventy-five days was selected based on the prolonged length of stay (LOS) observed in this cohort (maximum LOS 71.4 days).

### Statistical Analyses

Demographic variables, past medical history, clinical features, concomitant medication usage, hospital complications, and in-hospital outcomes were compared between COVID-19 patients who did or did not receive Zn + ionophore using the Mann-Whitney-U (Wilcoxon rank-sum) test for continuous variables and Chi-square test or Fisher’s exact test for categorical values, as appropriate.

A multivariable Cox proportional hazards model was fit for the time to in-hospital death using a time-dependent Zn + ionophore covariate to account for immortal time bias and the fact that medication usage with variable initiation times violated the proportional hazards assumption over the hospitalization period(17). This model was adjusted for confounders including age, sex, race, body mass index (BMI), diabetes, week of admission, hospital location, maximum sequential organ failure assessment (SOFA) score recorded during hospitalization, intubation/invasive mechanical ventilation, acute renal failure, neurological complications, treatment with corticosteroids, azithromycin or lopinavir/ritonavir and the propensity score for receiving Zn + ionophore. The propensity score was generated using logistic regression to model the predicted probability of receipt of Zn + ionophore as a function of age, sex, and ventilator status (as a marker of disease severity). The other covariates were selected based on known predictors of in-hospital death, biological plausibility and bivariate associations within our own data. Similar Cox regression models were fit for Zn alone, hydroxychloroquine alone, corticosteroids, azithromycin and lopinavir/ritonavir administration using the propensity score predicted probability specific to each medication and adjusting for the same covariates as the primary analysis. Subgroup analyses were conducted in dichotomized categories of age (dichotomized at the median), sex, ventilator status, corticosteroid, azithromycin and lopinavir/ritonavir use and forest plots were constructed. To determine if there was a difference in treatment effect for once versus twice daily zinc in addition to the ionophore, a Cox regression model was fit with a time-dependent covariate for twice daily dosing compared to a reference of once daily dosing, adjusting for the same covariates as the primary analysis, as well as the propensity score for once versus twice daily dosing (generated by logistic regression analysis adjusting for age, sex and ventilator status).

In a sensitivity analysis, a propensity score matched cohort was generated (matching for age, sex and ventilator status, as above) to reduce the imbalance in baseline characteristics between treatment groups when evaluating the treatment effects on the outcome of in-hospital mortality. In each propensity score-matched cohort, patients who did or did not receive Zn + ionophore were matched in a 1:1 ratio (random case order when drawing matches without replacement, with priority given to exact matches) with a 0.00003 propensity score matching radius. Similar Cox proportional hazard models were fit for the time to in-hospital death using a time-dependent Zn + ionophore covariate and adjusting for the same confounder covariates as the primary analysis. All analyses were conducted using IBM SPSS Statistics for Mac version 25 (IBM Corp., Armonk, NY).

## Results

**Figure 1:**
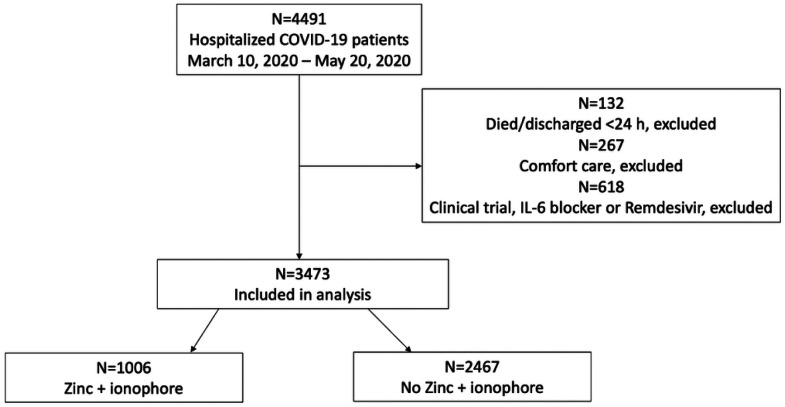
Flow chart of study inclusion

Between March 10 and May 20, 2020, 4,491 adult SARS-CoV-2 RT-PCR positive patients were hospitalized at our study sites. After excluding patients who died or were discharged within 24 hours, patients who received comfort measures only, those enrolled in clinical trials and those who received an IL-6 inhibitor or Remdesivir, 3,473 were included in analysis (Fig. 1). Of these 3,473 patients, 1,066 (29%) patients received Zn + ionophore beginning at a median of 0.5 days from admission. The median duration of treatment was three days (interquartile range interquartile range [IQR] 1.5–4.3 days). The median age was 64 years (IQR 50–76), 56% were male and 15% required invasive mechanical ventilation (Table 1). Patients who received Zn + ionophore were more often male, black, diabetic, had a higher BMI, were more often treated with corticosteroids and azithromycin, and less often treated with lopinavir/ritonavir compared to patients who did not receive Zn + ionophore (Table 2). In univariate analyses, rates of in-hospital mortality were significantly lower among patients who received Zn + ionophore compared to those who did not (12% died versus 17%, P < 0.001). Similarly, rates of discharge home were significantly higher among patients who received Zn + ionophore (72% versus 67% of patients who did not receive Zn + ionophore, P < 0.001).

**Figure 2:**
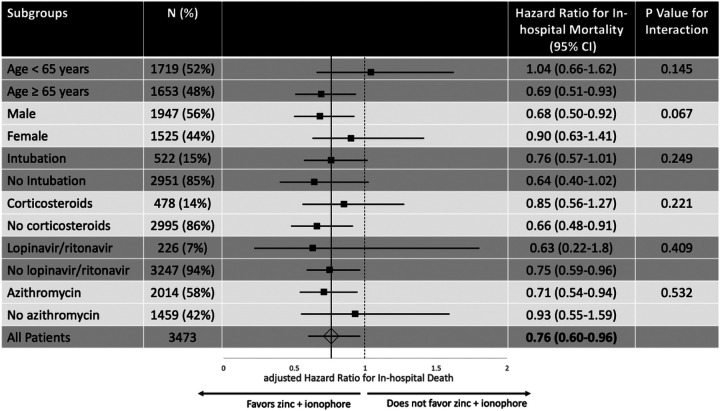
Forest plot of subgroups evaluating the impact of treatment with zinc plus an ionophore on in-hospital mortality

In Cox regression analysis adjusting for confounders, treatment with Zn + ionophore was associated with significantly reduced risk of in-hospital death (aHR 0.76, 95% CI 0.60–0.96, P = 0.023). Treatment with Zn alone (N = 1,097) did not affect mortality rates (aHR 1.14, 95% CI 0.89–1.44, P = 0.296) and treatment with the ionophore hydroxychloroquine alone (N = 2,299) appeared to be harmful (aHR 1.60, 95% CI 1.22–2.11, P = 0.001). In subgroup analysis, there were no significant interactions by age, intubation status, or use of other COVID-19 specific medications (corticosteroids, azithromycin, lopinavir/ritonavir), however, there was a suggestion of greater benefit of Zn + ionophore among males (Fig. 2). Among patients who received Zn + ionophore treatment, we did not identify a difference in treatment effect between once daily or twice daily dosing of zinc sulfate after adjusting for the same covariates used in the primary Cox regression analysis (aHR 0.90, 95% CI 0.56–1.45, P = 0.655). In similar adjusted Cox regression analyses, corticosteroid use (N = 478) was associated with higher rates of in-hospital mortality (aHR 2.2, 95% CI 1.74–2.69, P< 0.001). Among patient who received corticosteroids, 272/478 (57%) received methylprednisolone, 170/478 (36%) received prednisone and 78/478 (16%) received dexamethasone. The median time from admission to first dose of corticosteroids was 3 days (IQR 0.8–8.7 days) and the median duration of treatment was 4 days (IQR 1–7 days). There was no impact on in-hospital mortality for azithromycin (N = 2,014; aHR 1.22, 95% CI 0.99–1.51, P = 0.066) or lopinavir/ritonavir (N = 226; aHR 1.20, 95% CI 0.91–1.60, P = 0.204).

A sensitivity analysis in a propensity score-matched cohort produced consistent results. A total of 1,356 patients were included in this analysis (N = 678 Zn + ionophore, N = 678 No Zn + ionophore). The Zn + ionophore versus no Zn + ionophore groups were well matched for age, sex and intubation/invasive mechanical ventilation status (Supplemental Table 1). In this propensity score-matched cohort analysis, the median time from admission to Zn + ionophore administration was 0.6 days (IQR 0.3–0.9) and the median duration of treatment was 3 days (IQR 1.6–4.4). Similar to the primary analysis, Zn + ionophore was associated with a 37% reduced risk of in-hospital death (aHR 0.63, 95% CI 0.44–0.92, P = 0.015), while there was no significant association of in-hospital mortality with Zn alone (aHR 1.11,95% CI 0.77–1.60, P = 0.587) or the ionophore hydroxychloroquine alone (aHR 0.97, 95% CI 0.59–1.60, P = 0.897).

## Discussion

In this large cohort study with propensity score-matched sensitivity analysis, we evaluated the *a priori* hypothesis based on biologic plausibility, that treatment with zinc along with an ionophore (which increases intracellular zinc levels) would have a beneficial impact on patients with COVID-19. We found that Zn + ionophore was associated with a 24% relative risk reduction in rates of in-hospital death compared to patients who did not receive this medication combination. Neither zinc alone nor the ionophore alone (hydroxychloroquine) was associated with a reduction in mortality rates. In fact, hydroxychloroquine appeared to be harmful, though our primary analysis was not designed to evaluate the impact of hydroxychloroquine. The fact that a protective effect of Zn + ionophore was observed with fewer patients (Zn + ionophore N = 1,006) than the zinc alone group (N = 1,097) supports the biological hypothesis that an ionophore may be required to increase intracellular zinc levels to achieve therapeutic efficacy. Patients who received Zn + ionophore were also significantly more likely to be discharged home than those who did not. Hospital length of stay and ventilator days were significantly longer among patients who received Zn + ionophore, perhaps because in-hospital mortality rates were lower. The low cost of zinc sulfate ($0.05 /dose(21)) makes it an attractive therapeutic candidate compared to other drugs being evaluated for COVID-19 management such as remdesivir ($520.00 /dose(22)) or IL-6 blockers ($277.00 /600 mg dose of tocilizumab(23)). The cost of ionophores that are co-administered with zinc is similarly low (e.g. hydroxychloroquine: $4.36 /dose(24)).

Strengths of this study include the large sample size, and exclusion of comfort care patients or those who were dead or discharged within 24 hours, because these groups are less likely to receive COVID-19-specific medication and their inclusion could confound mortality analyses. Additionally, we excluded patients who received IL-6 inhibitors (tocilizumab, sarilumab, clazikuzumab) or remdesivir to avoid inadvertent observation of beneficial effect related to these agents. Similarly, patients enrolled in clinical trials were excluded because even patients randomized to a placebo group are treated differently than patients not participating in a trial. We did include patients who received corticosteroids, however, a variety of different steroid-class medications, doses and durations were utilized. Furthermore, this study was conducted prior to the RECOVERY trial(20), and use of corticosteroids was limited to severely hypoxic patients, which may explain the increased in-hospital mortality rates observed with corticosteroid use in this cohort. To limit the confounding impact of corticosteroids, we adjusted for their use in the multivariable Cox regression analyses. We also adjusted for the date of admission and hospital location to temper any effect that access to resources may have played in determining outcomes(25). Patients who received Zn + ionophore were actually enrolled earlier in the study window than those who did not, which could have biased this group toward worse outcomes due to resource scarcity, however, we still observed a beneficial effect. In addition to the above strengths, we also confirmed our results in a propensity score matched analysis, which controlled for baseline predictors of zinc use and in-hospital mortality.

There are limitations to this study. First, because this was an observational study, we are unable to fully account for all differences between patients who did or did not receive Zn + ionophore, despite adjusting for a variety of confounders in multivariable and propensity score-matched analyses. A number of randomized trials of zinc among COVID-19 patients are currently underway and should provide more definitive data. Second, we did not measure serum zinc levels prior to or after treatment, nor do we have any data on changes in intracellular zinc levels. It should be noted, however, that plasma zinc levels do not correlate well with tissue levels and active inflammation can acutely lower plasma zinc levels(26). Erythrocyte zinc concentrations may be a more accurate measure, but may not be readily available at many centers in a timely fashion. Additionally, the optimal duration of treatment with zinc is unknown. In our study, even a brief median duration of 3 days of Zn + ionophore use was associated with improved outcomes, though whether longer treatment would further improve outcomes or increase risk of zinc toxicity is unknown. We also did not have data on whether patients were taking zinc and/or hydroxychloroquine prior to admission. Zinc toxicity generally includes non-specific gastrointestinal symptoms, but prolonged exposure to high doses of zinc can lead to copper deficiency, which is associated with neurological abnormalities including myeloneuropathy and cognitive deficits(27), as well as hepatosplenomegaly and osteoporosis. Third, since only adults were included in this study, our results cannot be generalized to children. Fourth, hydroxychloroquine was used as the zinc ionophore out of expediency, since at the time of the study, hydroxychloroquine had hypothetical benefit in COVID-19(28, 29), in addition to functioning as a supporting agent to promote increased intracellular zinc levels. The use of hydroxychloroquine in COVID-19 has been controversial and recent randomized, controlled trials have not supported its use(30) and have identified significant risk of prolonged QTc interval and elevated transaminases(30). Alternative ionophores such as hinokitiol(8), resveratrol(9) (found in red wine), quercetin (a flavonoid found in red wine, kale, onions, green tea) or epigallocatechin-gallate(10) (found in green tea) might prove useful, but little data currently exists supporting their use in COVID-19 patients.

## Conclusions

This is the first large cohort study to evaluate the impact of zinc on outcome in hospitalized COVID-19 patients. Our results demonstrate a significant reduction in hospital morality rates when zinc is combined with an ionophore to maximize intracellular zinc levels. The low cost of zinc, compared to other treatments such as remdesivir or IL-6 blockers, and its favorable side effect profile, make zinc an attractive therapeutic option. Randomized trials of zinc and exploration of alternate ionophores are merited.

## Supplementary Material

Supplement

## Figures and Tables

**Table 1:** Cohort characteristics of patients who received zinc with an ionophore

Variable	Hospitalized Adult Patients from March 10, 2020-May 20, 2020, N = 3473
Zinc + Ionophore-N (%)	1006 (29%)
Days from admission to first dose-median (IQR)	0.5 (0.3–0.9)
Duration of treatment (days)-median (IQR)	3.0 (1.5–4.3)
Total dose of zinc gluconate (elemental zinc, mg)-median (IQR)	1100 mg (250 mg elemental zinc), (660–1980 mg)
Age- median (IQR)	64 (50–76)
Male sex-N (%)	1947 (56%)
Race- N (%)	1600 (46%)
White	570 (16%)
Black	225 (7%)
Asian	1078 (31%)
Other/unknown	
Admission Date- median (IQR)	April 3, 2020 (March 27-April 14, 2020)
Body Mass Index-median (IQR)	28 (24–33)
Invasive mechanical ventilation -N (%)	522 (15%)
Maximum SOFA score during hospitalization-median (IQR)	3 (0–4)
In-hospital mortality-N (%)	545 (16%)
Discharge home-N (%)	2338 (67%)

IQR = intraquartile range, SOFA = sequential organ failure assessment

**Table 2:** Demographic and clinical characteristics of patients who received zinc with an ionophore (N = 3473)

Characteristic	Zinc + Ionophore N = 1006	No Zinc + Ionophore N = 2467	P
**Demographics**			
Median Age (IQR)-yr	64 (53–74)	64 (49–76)	0.379
Male sex-N (%)	604 (60%)	1344 (55%)	0.003
Race- N (%)	409 (40%)	1103 (45%)	0.010
White	196 (20%)	365 (15%)	
Black	67 (7%)	149 (6%)	
Asian	334 (33%)	850 (34%)	
Other/unknown			
Body Mass Index-median (IQR)	29 (25–33)	28 (24–33)	0.001
Past Medical History- N (%)	387 (39%)	934 (39%)	0.737
Hypertension	289 (29%)	622 (25%)	0.033
Diabetes			
**Clinical Features**			
Date of Admission-median (IQR)	April 2, 2020 (March 30-April 9, 2020)	April 3, 2020 (March 26-April 18, 2020)	0.778
Maximum SOFA score- median (range)	3 (0–21)	3 (0–23)	<0.001
**Concomitant COVID-19 specific Medications**			
Corticosteroids- N (%)	190 (19%)	288 (12%)	<0.001
Azithromycin-N (%)	847 (84%)	1167 (47%)	<0.001
Lopinavir/ritonavir-N (%)	40 (4%)	186 (8%)	<0.001
**Hospital complications**			
Neurological Event- N (%)	142 (14%)	378 (15%)	0.366
Mechanical Ventilation- N (%)	162 (16%)	360 (15%)	0.258
Acute renal failure- N (%)	129 (13%)	291 (12%)	0.400
**Outcomes**			
Died in-hospital- N (%)	121 (12%)	424 (17%)	<0.001
Discharged home- N (%)	715 (72%)	1623 (67%)	0.003
Other Discharge Dispositions, N (%)	7 (1%)	12 (1%)	0.454
Hospitalized	8 (1%)	23 (1%)	0.843
LTACH	120 (12%)	292 (12%)	0.997
Nursing home	25 (3%)	49 (2%)	0.372
Acute inpatient rehabilitation	1 (0.1%)	5 (0.2%)	0.502
Subacute rehabilitation			
Hospital length of stay (days)-median (IQR)	6.8 (4.0–11.0)	4.9 (2.8–8.4)	<0.001
Ventilator days^¶^-median (IQR)	7.4 (1.4–19.7)	4.1 (1.2–8.9)	0.003

HCQ = hydroxychloroquine, IQR = intraquartile range, SOFA = sequential organ failure assessment; LTACH = long term acute care hospital,

¶ =includes invasive mechanical ventilation only

## Abbreviations

COVID-19: coronavirus disease 2019
Zn+ionophore: zinc with an ionophore
RT-PCR: reverse-transcriptase-polymerase-chain-reaction
SOFA: sequential organ failure assessment
aHR: adjusted Hazard Ratio
EMR: electronic medical record
COPD: chronic obstructive pulmonary disese
LOS: length of stay
BMI: body mass index (BMI)
IQR: inter quartile range
IL-6: interleukin-6
