# Supplementary material for: Treatment with Zinc is Associated with Reduced In-Hospital Mortality Among COVID-19 Patients: A Multi-Center Cohort Study
Source: Res Sq. 2020 Oct 26:rs.3.rs-94509. Preprint. [Version 1] doi: 10.21203/rs.3.rs-94509/v1 (PMC7605567; doi:10.21203/rs.3.rs-94509/v1)
Supplement: Supplement [file 437e4058ac3eb7b1add507a4.docx]

**Supplementary Appendix**

This appendix has been provided by the authors to give readers additional information about their work.

**TABLE OF CONTENTS**

**Methods** Pages 3-4

**Table S1** Pages 5-6

**METHODS:**

*Medication Utilization-*

Hospital guidelines for the treatment for COVID-19 patients (instituted in March, 2020) recommended administration of hydroxychloroquine (400 mg BID for one day then 200 mg BID for four days) +/- azithromycin (500 mg daily for one day then 250 mg daily for four days) in patients whose oxygen saturation was <94% on room air and whose QTc interval was <500 ms. Among patients receiving both hydroxychloroquine and azithromycin, a daily EKG was recommended along with discontinuation of all other Qtc prolonging medications. Azithromycin was considered contraindicated in patients with Qtc>500 ms. Among patients with a prolonged Qtc (>500 ms) who were not receiving medications that could interfere with metabolism (e.g. cytochrome P450 inhibitors/inducers), lopinavir/ritonavir (400 mg/100 mg PO twice daily for five days) was recommended. Lopinavir/ritonavir was also recommended as first line treatment for patients with ≤ 3 days of symptoms who were at risk for disease progression (e.g. age≥60 years, BMI≥30, malignancy, immunocompromised, chronic lung, kidney or liver disease, and/or poorly controlled diabetes or hypertension). Anticoagulation was continued during hospitalization in patients with an underlying indication for anticoagulation. In patients without a prior known indication, serial D-Dimer levels were checked every 48 hours and therapeutic anticoagulation (monitoring heparin or enoxaparin specific Xa levels) was recommended in patients with D-dimer levels >10,000 ng/mL and suggested in those with D-dimer levels between 2,000-10,000 ng/mL. Prophylactic dosing of antithrombotics was recommended for patients with D-dimer levels <2000 ng/mL. The preferred agent for both prophylactic and therapeutic anticoagulation was enoxaparin, though intravenous heparin was preferred in patients with multisystem organ failure, acute respiratory failure requiring invasive mechanical ventilation or in patients with rapidly progressing renal failure. In patients without a confirmed indication for anticoagulation, antithrombotics were recommended for the duration of hospitalization. In patients with high suspicion for a venous thromboembolic event, oral anticoagulation could be considered for at least 3-months following discharge.

Revisions to the guideline in April 2020, removed azithromycin and nitazoxamide from the algorithm after identification of significant QTc prolongation with azithromycin use. Tocilizumab (400 mg IV once) or Anakirnra (200 mg SQ daily) could be considered in hypoxic patients (requiring at least 4-6 L O2). Remdesivir could be obtained as part of a clinical trial or via an expanded access program for intubated patients who were not receiving vasopressor agents and with a glomerular filtration rate >30. Other IL-6 blockers (Sarilumab or Clazakiszumab) and convalescent plasma were available only as part of clinical trials.

**Supplemental Table 1.** Characteristics of the Propensity Score-Matched Cohort N=1356

| **Characteristic** | **Zinc + Ionophore**  **N=678** | **No Zinc + Ionophore**  **N=678** | **P** |
| --- | --- | --- | --- |
| **Demographics** |  |  |  |
| Median Age (IQR)-yr | 65 (55-74) | 65 (54-75) | 0.883 |
| Male sex-N (%) | 402 (59%) | 409 (60%) | 0.698 |
| Race- N (%)  White  Black  Asian  Other/unknown | 288 (43%)  132 (20%)  47 (7%)  211 (30%) | 301 (44%)  102 (15%)  41 (6%)  234 (35%) | 0.332 |
| Body Mass Index-median (IQR) | 29 (25-34) | 28 (24-33) | 0.049 |
| Past Medical History- N (%)  Hypertension  Diabetes | 275 (41%)  196 (29%) | 276 (41%)  184 (27%) | 0.956  0.468 |
| **Clinical Features** |  |  |  |
| Date of Admission-median (IQR) | April 3, 2020  (March 30-April 9, 2020) | April 2, 2020  (March 25-April 16, 2020) | 0.047 |
| Hospital complications- N (%)  Neurological Event  Mechanical Ventilation  Acute renal failure | 104 (15%)  104 (15%)  84 (12%) | 84 (12%)  88 (13%)  81 (12%) | 0.116  0.213  0.803 |
| Maximum SOFA score- median (range) | 3 (0-21) | 3 (0-18) | <0.001 |
| **Concomitant COVID-19 specific Medications** |  |  |  |
| Corticosteroids- N (%) | 131 (19%) | 76 (11%) | <0.001 |
| Azithromycin-N (%) | 568 (84%) | 337 (50%) | <0.001 |
| Lopinavir/ritonavir-N (%) | 26 (4%) | 58 (9%) | <0.001 |
| **Outcomes** |  |  |  |
| Died in-hospital- N (%) | 81 (12%) | 109 (16%) | 0.028 |
| Discharged home— N (%) | 476 (35%) | 466 (35%) | 0.446 |
| Hospital length of stay (days)—median (IQR) | 6.7 (4.1-10.9) | 4.9 (2.9-8.0) | <0.001 |
| Ventilator days^¶^-median (IQR) | 7.8 (1.3-18.1) | 4.2 (1.4-8.4) | 0.014 |
| Other Discharge Dispositions, N (%)  Hospitalized  LTACH  Nursing home  Acute inpatient rehabilitation  Subacute rehabilitation | 4 (0.6%)  4 (0.6%)  87 (13%)  19 (3%)  0 (0%) | 3 (0.4%)  5 (0.7%)  73 (11%)  13 (2%)  1 (0.1%) | 0.705  0.738  0.238  0.283  0.317 |

HCQ=hydroxychloroquine, IQR=intraquartile range, SOFA=sequential organ failure assessment; LTACH=long term acute care hospital.
